# Subthalamic and Cortical Local Field Potentials Associated with Pilocarpine-Induced Oral Tremor in the Rat

**DOI:** 10.3389/fnbeh.2016.00123

**Published:** 2016-06-17

**Authors:** Lauren L. Long, Samantha J. Podurgiel, Aileen F. Haque, Emily L. Errante, James J. Chrobak, John D. Salamone

**Affiliations:** Department of Psychological Sciences, University of ConnecticutStorrs, CT, USA

**Keywords:** Parkinsonism, acetylcholine, muscarinic, motor cortex, LFP, tremulous jaw movements

## Abstract

Tremulous jaw movements (TJMs) are rapid vertical deflections of the lower jaw that resemble chewing but are not directed at any particular stimulus. In rodents, TJMs are induced by neurochemical conditions that parallel those seen in human Parkinsonism, including neurotoxic or pharmacological depletion of striatal dopamine (DA), DA antagonism, and cholinomimetic administration. Moreover, TJMs in rodents can be attenuated by antiparkinsonian agents, including levodopa (L-DOPA), DA agonists, muscarinic antagonists, and adenosine A_2A_ antagonists. In human Parkinsonian patients, exaggerated physiological synchrony is seen in the beta frequency band in various parts of the cortical/basal ganglia/thalamic circuitry, and activity in the tremor frequency range (3–7 Hz) also has been recorded. The present studies were undertaken to determine if tremor-related local field potential (LFP) activity could be recorded from motor cortex (M1) or subthalamic nucleus (STN) during the TJMs induced by the muscarinic agonist pilocarpine, which is a well-known tremorogenic agent. Pilocarpine induced a robust TJM response that was marked by rhythmic electromyographic (EMG) activity in the temporalis muscle. Compared to periods with no tremor activity, TJM epochs were characterized by increased LFP activity in the tremor frequency range in both neocortex and STN. Tremor activity was not associated with increased synchrony in the beta frequency band. These studies identified tremor-related LFP activity in parts of the cortical/basal ganglia circuitry that are involved in the pathophysiology of Parkinsonism. This research may ultimately lead to identification of the oscillatory neural mechanisms involved in the generation of tremulous activity, and promote development of novel treatments for tremor disorders.

## Introduction

Parkinsonism is a broad family of disorders that includes idiopathic Parkinson’s disease (PD), which results from degeneration of nigrostriatal dopamine (DA) neurons (Hornykiewicz, 1973), and drug-induced Parkinsonism, which is induced by drugs that interfere with DA transmission (e.g., DA antagonists, DA depleting agents; Marsden et al., 1975; McEvoy, 1983), and cholinomimetics such as anticholinesterases and muscarinic agonists (Ott and Lannon, 1992; Aarsland et al., 2003). The cardinal motor symptoms of Parkinsonism include akinesia, bradykinesia, rigidity, and a resting tremor that typically occurs in the 3–7 Hz frequency range (Marsden et al., 1975). Motor dysfunctions resembling drug-induced Parkinsonism are produced in rodents by the same pharmacological agents that induce human Parkinsonism, and resting tremor can be modeled in rodents using the tremulous jaw movement (TJM) model. TJMs, which are defined as “rapid, repetitive vertical deflections of the lower jaw that resemble chewing but are not directed at any particular stimulus” (Salamone et al., 1998), have a local frequency of 3–7 Hz, which parallels that of Parkinsonian resting tremor (Cousins et al., 1998; Salamone et al., 1998, 2015a; Collins et al., 2010; Podurgiel et al., 2013b). TJMs are induced by neurotoxic or pharmacological depletion of striatal DA, DA antagonism, and cholinomimetic drugs (Jicha and Salamone, 1991; Salamone et al., 1998; Podurgiel et al., 2013a,b, 2015; Gandía et al., 2015), and can be attenuated by co-administration of antiparkinsonian agents such as levodopa (L-DOPA), DA agonists, muscarinic antagonists, MAO inhibitors, and adenosine A_2A_ antagonists (Cousins et al., 1997; Simola et al., 2004, 2006; Salamone et al., 2005, 2008a,b, 2015a,b; Podurgiel et al., 2013a,b). Considerable evidence indicates that TJMs in rodents are a valid model for the exploration of the pharmacology, neurochemistry and physiology of drug-induced tremor (Salamone et al., 1998, 2008b, 2013, 2015a; Collins-Praino et al., 2011; Podurgiel et al., 2013a,b, 2015; Herrera-Meza et al., 2014; Gandía et al., 2015).

One method that can be used for studying the neural mechanisms underlying tremorogenesis is to record fluctuations in the electrical activity of the brain, such as local field potentials (LFPs). Measures of LFP activity provide a way to characterize the synchronous oscillatory neural activities that are potentially related to motor dysfunctions. Exaggerated neuronal synchrony has been recorded in basal ganglia and cortex of PD patients, with the beta band (~15–30 Hz) being the best-characterized oscillation (Brown, 2003; Hammond et al., 2007; Oswal et al., 2013). Increased beta activity has been observed in the cortex (George et al., 2013) and subthalamic nucleus (STN) of PD patients, and it has been suggested that excessive synchrony in this frequency range contributes to motor dysfunction (Levy et al., 2002; Brown and Williams, 2005; Kühn et al., 2006). Reductions in STN beta activity correlate with improvements in akinesia and rigidity in PD patients (Levy et al., 2002; Kühn et al., 2006). While the literature supports a link between increased cortical and basal ganglia beta power and the development of akinesia/rigidity, beta activity generally does not correlate with the severity of resting tremor (Kühn et al., 2005; Hammond et al., 2007; Oswal et al., 2013). Rather, the development of tremor in PD patients was shown to be associated with the emergence of oscillations in the tremor frequency range (3–7 Hz) in the cortex and basal ganglia (Timmermann et al., 2003; Reck et al., 2009; Hirschmann et al., 2013; Oswal et al., 2013). Timmermann et al. (2003) reported strong coherence between electromyograph (EMG) activity of forearm muscles and activity in the contralateral primary motor cortex (M1), at tremor (3–7 Hz) and double tremor frequency (7–13 Hz) in PD patients off medication. Similar patterns of activity have been observed in the STN of PD patients, revealing power spectra peaks at tremor frequency and tremor harmonics, as well as significant coherence between STN LFPs and EMG activity at tremor frequency (Levy et al., 2000; Brown et al., 2001; Liu et al., 2002; Wang et al., 2005; Reck et al., 2009).

These clinical reports support the idea that cortical and STN power and coherence at tremor frequencies increases with the manifestation of tremor, but this phenomenon has not been modeled extensively in rodents. Therefore, the present study characterized the temporal pattern of oral EMG activity and associated changes in LFPs recorded from M1 and STN during the TJMs induced by the muscarinic agonist pilocarpine. Pilocarpine was selected for these studies because our laboratory has a large data base on the pharmacology of cholinomimetic-induced TJMs, including studies demonstrating that these movements can be attenuated by co-administration of antiparkinsonian drugs (Salamone et al., 1998, 2013; Collins et al., 2010; Collins-Praino et al., 2013).

## Materials and Methods

### Animals

A total of five adult male Sprague Dawley rats (Harlan Sprague Dawley, Indianapolis, IN, USA) with no prior drug experience were used in the present experiment. The rats weighed 350–450 g during the course of the experiment and had *ad libitum* access to lab chow and water. Animals were group-housed prior to surgery in a colony that was maintained at approximately 23°C and had a 12-h light/dark cycle (lights on at 0700 h). Post-surgery, animals were single housed to avoid over grooming around the surgical implant.

### Drug Treatment Procedures and Dose Selection

Pilocarpine was purchased from Sigma Aldrich Chemical (St. Louis, MO, USA) and dissolved in 0.9% saline. The dose of pilocarpine (4.0 mg/kg) was based on previous experiments showing significant induction of jaw movements at this dose (for further details, see Collins et al., 2010).

### Surgical Procedures

#### Ethics Statement

All procedures performed were in strict accordance with the guidelines and regulations implemented by the University of Connecticut’s Institutional Animal Care and Use Committee and NIH. The protocol was approved by the Institutional Animal Care and Use Committee of the University of Connecticut (Protocol Number: A15–014) and all efforts were made to minimize suffering.

Rats were anesthetized with a 1.0 ml/kg IP injection of a cocktail solution containing 10.0 ml of 100 mg/mL ketamine plus 0.75 ml of 20.0 mg/ml xylazine (Phoenix Scientific, Inc., St. Joseph, MO, USA). Rats were placed in a stereotaxic frame (Kopf, Tujunga, CA, USA), and a midline scalp incision was made. Two electrode arrays consisting of 50 μm tungsten wire (California Fine Wire Company, Grover Beach, CA, USA) were bilaterally implanted with a 27-gauge needle approximately 5.0 mm deep into the lateral temporalis muscle (four EMG electrodes per animal). Previous research has demonstrated that the lateral temporalis muscle is the jaw muscle that shows activity most closely related to TJMs (Cousins et al., 1998). Burr holes were drilled through the skull over the STN (R hemisphere) and M1 (L hemisphere), and 2–4 electrode arrays were implanted (eight LFP electrodes per animal). LFP electrode arrays were comprised of four linearly spaced 50 μm tungsten wires (California Fine Wire Company, Grover Beach, CA, USA). Electrode wire was arranged and separated by fused silica tubing (Polymicro Tubing, Phoenix, AZ, USA), attached to female pins (Omnetics, Minneapolis, MN, USA) and secured in a rectangular five by four pin array. Two stainless steel watch screws driven into the skull above the cerebellum served as indifferent and ground electrodes. Supplementary anchor screws were positioned as necessary and the entire head-stage ensemble was fortified with dental acrylic. The surgical coordinates, for which Bregma and the top of the skull was used as the reference point, were as follows: STN (AP: −3.6, ML: ± 2.5, DV: −7.5); M1 (AP: +1.0, ML: +1.9, DV: −2.5). Rats recovered for 1 week post-surgical procedure.

### Behavioral Measures

Following a one-week recovery period, rats were given an acute IP injection of saline (vehicle). Immediately after vehicle injection, rats were placed into a Plexiglas observation chamber and allowed to habituate for 10 min. At the beginning of this habituation period, the animals were connected to the recording apparatus by a multi-channel tether (Neuralynx, Bozeman, MT, USA) that was attached to a pulley system in the ceiling. Following the habituation period, a trained observer counted TJMs for 15 min. TJMs were defined as rapid vertical deflections of the lower jaw that resembled chewing but were not directed at any particular stimulus (Salamone et al., 1998). At the end of the 15-min observation period, rats were disconnected and returned to their home cages. This procedure was repeated with administration of 4.0 mg/kg pilocarpine 24 h later.

### Electrophysiological Data Acquisition and Analysis

Following the habituation period, wide-band electrical activity was recorded (5050.5 samples/s) for 15 min using a Neuralynx data acquisition system (Bozeman, MT, USA). TJMs were counted simultaneously by a trained observer and noted using event markers through Cheetah data acquisition software (version 5.6.3; Neuralynx, Bozeman, MT, USA). Following data acquisition and during subsequent offline analysis, data were imported into Matlab R2014a (Mathworks, Natick, MA, USA).

The raw EMG signal was bandpass filtered between 500 and 1500 Hz and the Hilbert transform, which provides an analytic representation of the signal, was computed on the bandpass filtered signal using Matlab. In this regard, the instantaneous (5050.5 samples/s) EMG envelope amplitude (magnitude of Hilbert transform) was obtained over time. The EMG signal was then full wave rectified, to generate the absolute value of signal, and all raw EMG traces presented in the current analysis represent this full wave rectified signal. Event markers (as denoted in Cheetah data acquisition software) were simultaneously imported into Matlab and plotted along with the EMG signal. The presence or absence of these event markers was used to identify TJM and no TJM epochs, respectively. Raw LFP data were imported into Matlab R2014a and down-sampled by a factor of 10 during offline analysis, thus changing the sampling rate to 505.05 samples/s (Hz; 5050.5/10 = 505.05). The raw LFP signal was lowpass filtered to remove high frequency chewing, chattering and/or teeth grinding artifacts (*F*_c_ = 250 Hz). Then, the LFP signal was bandpass filtered for tremor (3–7 Hz) and beta frequency (15–30 Hz) and the Hilbert transform was computed on the bandpass filtered signals. Data were examined during: (1) TJM epochs; and (2) No TJM epochs.

All data analysis was conducted using custom written programs in MatLab R2014a (Mathworks, Natick, MA, USA). Power spectral density estimates were obtained using Welch’s averaged modified periodogram method (Welch, 1967) during epochs of TJMs and no TJMs. To extract the amplitude modulation (AM) frequency of the EMG signal (e.g., “tremor frequency”, 3–7 Hz), power spectral density estimates were obtained from the envelope (magnitude of Hilbert transform) of the bandpass filtered EMG signal. For LFP data, the power for tremor (3–7 Hz) and beta frequency (15–30 Hz) was calculated from the bandpass filtered signals and represented in units of mV^2^. Average power was calculated by taking the sum of the power values within a given frequency range of interest (e.g., 3–7 Hz) and multiplying the sum by the spectral window resolution. LFP centroid frequency (i.e., the weighted mean frequency) was calculated by first isolating the frequency range of interest (e.g., 3–7 Hz), indexing the power values within that frequency range and multiplying them. Then, we divided those values by the sum of the previously indexed power values. Lastly, we took the sum of the aforementioned (see for formula below for calculation of centroid frequency).

(1) $$C\;=\;\frac{\sum_{n\;=\;0}^{N-1}f(n)x(n)}{\sum_{n\;=\;0}^{N-1}x(n)}$$

### Statistical Analyses

Comparisons between the total number of TJMs observed after injections of vehicle and pilocarpine were performed using the repeated measures *t*-test. Although vehicle conditions were used to verify the ability of pilocarpine to produce TJMs, large differences existed between vehicle and pilocarpine recordings that made electrophysiological comparisons between these two signals unsuitable. Essentially, vehicle treatments produce no tremor bursts, but more movement artifact. Because of these differences, all EMG and LFP data analysis were conducted on epochs of TJMs and no TJMs within the pilocarpine recording for each animal. For each of the five animals a representative 2.2 s TJM was identified by a trained observer using event markers. The TJM length was determined by the lowest responding animal. Further, within the same recording for each animal a subsequent 2.2 s no TJM time point was isolated as indicated by lack of event markers. For each electrode, the average LFP power and centroid frequency was computed within the frequency range of interest (3–7 Hz and 15–30 Hz) for TJM and no TJM epochs all the while discretized by electrode location (M1 and STN). Paired samples *t*-tests were computed to asses if there were significant differences in: (1) average tremor LFP power during TJMs and no TJMs for M1; (2) average tremor LFP power during TJMs and no TJMs for STN; (3) average beta LFP power during TJMs and no TJMs for M1; (4) average beta LFP power during TJMs and no TJMs for STN; and (5) the same as 1–4, but for centroid frequency. Further, we computed paired-samples *t*-tests to examine if there were differences in the aforementioned indices as a function of brain area (M1 and STN). Lastly, paired samples *t*-tests were used to confirm significant differences in number of TJMs per 15 min recording period across vehicle and pilocarpine recordings.

### Histology

At the completion of the experiment, animals were deeply anesthetized with CO_2_ and perfused with 0.9% physiological saline followed by 3.7% formaldehyde solution. The brains were extracted and stored in the formaldehyde solution for 1 week. Then, brains were sliced (50 μm sections) using a vibratome (Leica, Germany), mounted, Nissl stained using Cresyl Violet and cover-slipped allowing for verification of electrode placements. Photomicrographs of electrode tracks were taken using a Nikon microscope connected to a Spot RT camera system, digitized and prepared for presentation using Adobe Photoshop. Consistent with the histological criteria employed by Brown et al. (2011) only placements that were within 500 μm of the STN, but dorsal to the cerebral peduncles and internal capsule were used for statistical analyses.

## Results

### Histological Verification of Electrode Placements

**Figure 1:**
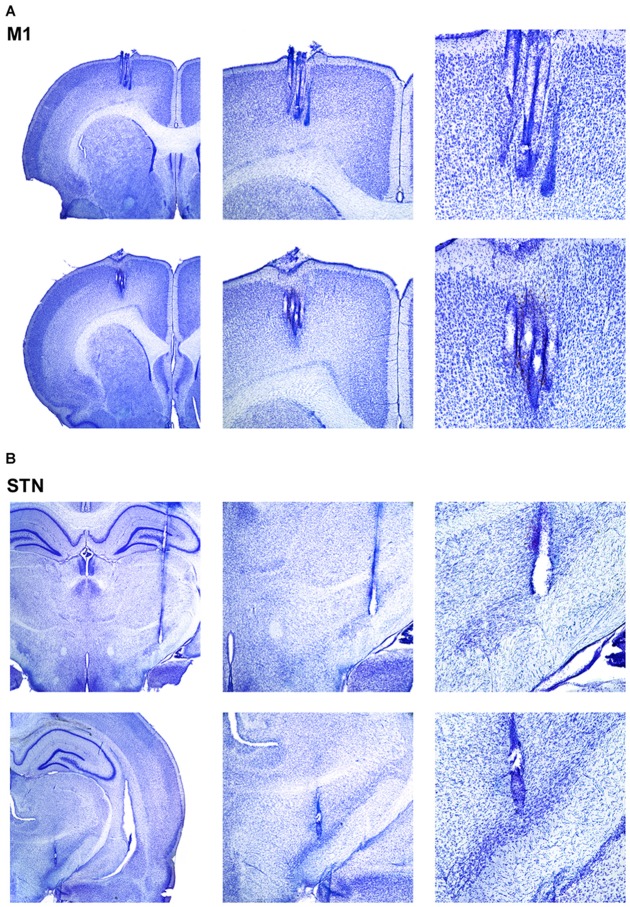
**Verification of electrode placements.** (**A**, top): Photomicrographs of four representative and simultaneously recorded sites in motor cortex (M1). Middle and right photomicrographs show 4× and 10× close-up of electrode tips, respectively. (**A**, bottom): Same as (**A**, top) but for a different animal. (**B**, top): Photomicrographs of a representative recording site in subthalamic nucleus (STN). Middle and right photomicrographs show a 4× and 10× close-up of electrode tips, respectively. (**B**, bottom): Same as (**B**, top) but for a different animal.

A total of 38 LFP electrodes (*n* = 19 M1 electrodes; *n* = 19 STN electrodes) and five EMG electrodes across five animals were used in the current analysis (see Supplementary Figure S1 for histology across all animals; atlas templates modified from Paxinos and Watson, 2014). All animals contributed one EMG electrode, 3–4 M1 (Figure 1A) and 3–4 STN (Figure 1B) electrodes. All EMG and LFP data were simultaneously recorded from each animal. M1 electrodes terminated in all cortical layers but were more likely to terminate in deeper layers. Further, most STN electrodes were hits, although a few terminated slightly dorsally or anteriorly (data not shown), but still within the 500 μm criteria put forth by Brown et al. (2011).

### Pilocarpine Induces TJMs in the Tremor Frequency Range (3–7 Hz) as Reflected by EMG

**Figure 2:**
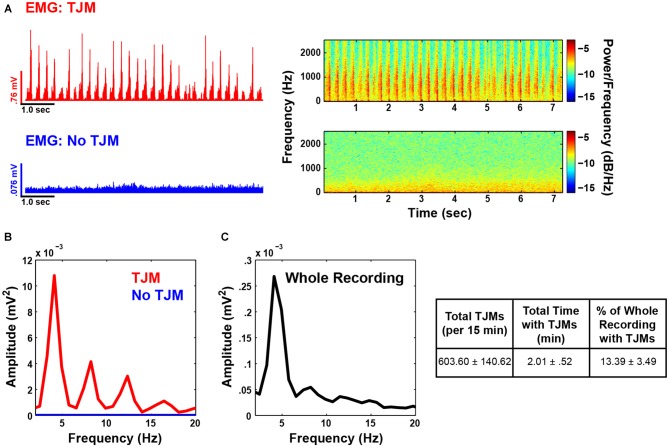
**Pilocarpine induces Tremulous jaw movements (TJMs) in the tremor frequency range as reflected by electromyograph (EMG) activity.** (**A**, top): EMG electrode trace bandpass filtered for EMG frequency (500–1500 Hz) and full wave rectified during a long epoch of TJMs (red; 7.3 s) for a representative animal. The spectrogram (right) indicates the same pattern of rhythmic activity as the EMG trace. (**A**, bottom): Same animal and recording time point as presented in **(A)**, but for a long period of quiescence (blue; 7.3 s). The spectrogram of raw and unfiltered signal (right) indicates little rhythmic activity during bouts lacking TJMs. **(B)** Power spectrum of the EMG envelope (i.e., a smoothed curve outlining the extremes of the signal) for the same traces as presented in **(A)**. As can be seen, there is clear 3–7 Hz rhythmicity and strong harmonics during TJM epochs (red), which is absent during periods of quiescence (blue). (**C**, left): Power spectrum of the entire pilocarpine recording (sans TJM and no TJM epoch isolation) for the same animal as presented in **(A,B)**. (**C**, right): Behavior summarized across all animals for the entirety of the pilocarpine recording.

Administration of pilocarpine significantly induced TJMs compared to vehicle control (Mean ± SEM; Pilo: 603.6 ± 140.62; Vehicle: 14.4 ± 4.4; *t*_(4)_ = 4.3, *p* < 0.05). As described above, all EMG and LFP data analysis were conducted on epochs of TJMs vs. no TJMs within the pilocarpine recording for each animal. During bouts of jaw movement activity, the bandpass filtered (500–1500 Hz) and full wave rectified EMG signal was marked by rhythmic activity in the 3–7 Hz range (Figure 2A top, left; red). Conversely, during periods of quiescence, no rhythmic activity was observed in the EMG (Figure 2A bottom, left; blue). Spectrograms of the raw and unfiltered signals indicated the same pattern of activity as the filtered EMG traces (Figure 2A, right). For the traces presented in Figure 2A, power spectral analysis revealed strong rhythmicity in the envelope of the EMG signal with a fundamental frequency of 4 Hz along with robust 2nd and 3rd harmonics (Figure 2B, red). Epochs of quiescence failed to exhibit AM of the EMG signal within the tremor frequency range (Figure 2B, blue) and show relatively little power overall. Upon examination of the entire pilocarpine recording for the same representative animal, rodents continued to exhibit dominant power of the EMG envelope within the tremor frequency range, although the harmonics were largely attenuated (Figure 2C). On average, animals spent 13.39% of the 15-min recording session exhibiting TJMs (Figure 2C, right) indicating the robustness of the observed phenomenon.

### Power in the Tremor Frequency Band Increases during TJMs in M1 and STN

**Figure 3:**
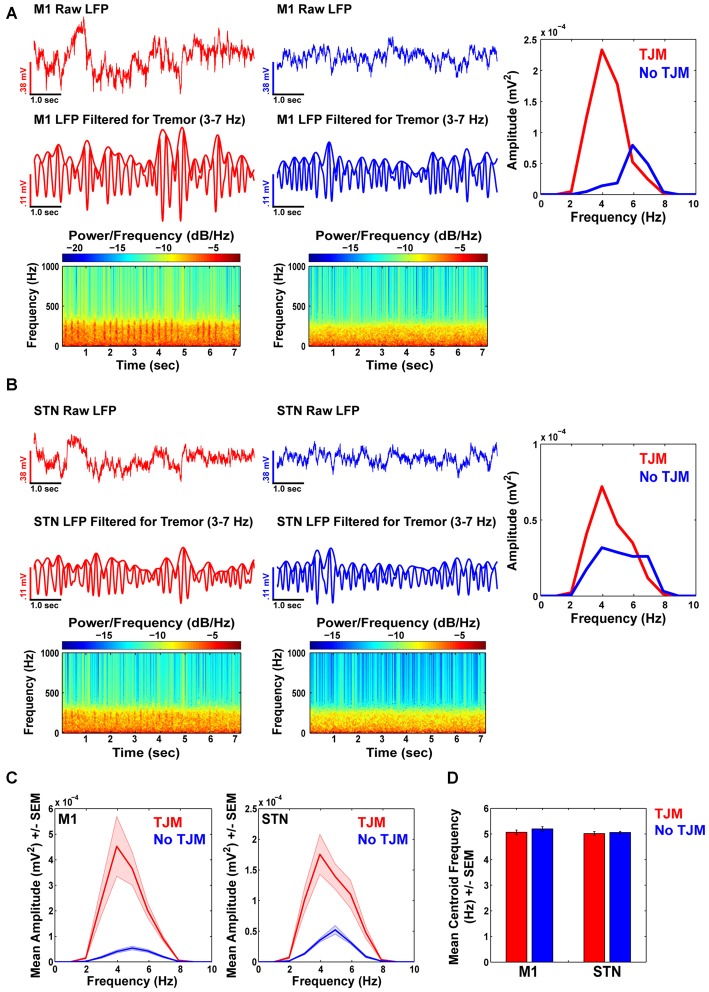
**Power in tremor frequency band increases during TJMs in M1 and STN. (A)** Raw (top) and bandpass filtered (3–7 Hz; middle) M1 LFP traces during epochs of TJMs (red) and no TJMs (blue) for the same representative animal presented in Figure 2. (**A**, bottom): Corresponding spectrograms for the raw M1 signal presented in the top panel of **(A)** during TJM and no TJM epochs. Overall, the spectrograms of raw and unfiltered signal indicate rhythmicity in the LFP during periods of TJMs that is lacking during periods of quiescence. (**A**, right): Power spectrum of the bandpass filtered LFP signal presented in the middle panel of **(A)** during the TJM (red) and no TJM (blue) epoch. Tremor frequency power increases substantially during periods of TJMs, while little tremor power exists during epochs of quiescence. **(B)** Same as **(A)**, but for a simultaneously recorded STN site for the same representative animal presented in **(A). (C)** Summary data for tremor frequency power across all animals for TJM and no TJM epochs for M1 and STN. As can be seen, tremor band power dominates during the presence of TJMs, but not for bouts lacking TJMs for both M1 and STN recording sites. **(D)** Summary data across all animals for tremor band centroid frequency as a function of TJM and no TJM epochs for M1 and STN recording sites. Overall, there were no differences in centroid frequency across behavioral state or brain area.

Upon examination of simultaneously recorded M1 LFPs for the same animal as in Figure 2, the raw (Figure 3A top) and bandpass filtered (3–7 Hz; Figure 3A middle) LFP signals revealed increased power in the tremor frequency band during epochs of TJMs (Figure 3A left, red), but not during bouts of quiescence (Figure 3A right, blue). The LFP signal was indexed during epochs of TJMs and no TJMs as indicated by EMG event markers or lack thereof, respectively. Spectrograms of the raw and unfiltered signal revealed differential patterns of LFP activity for bouts of TJMs and quiescence (Figure 3A bottom). The power spectrum of the raw LFP signal filtered for tremor frequencies (3–7 Hz) during epochs of TJMs (Figure 3A right, red) and no TJMs (Figure 3A right, blue) indicated strong LFP power in the tremor range with a peak at ~4 Hz for bouts of TJMs, while little LFP power existed at such frequencies for bouts lacking TJMs. At a simultaneously recorded STN site, the same pattern of activity was present (Figure 3B). Overall, the bandpass filtered LFP signal (3–7 Hz) during bouts of TJMs and the subsequent power spectrum of that signal revealed strong power in the 3–7 Hz range, whereas bouts of quiescence did not exhibit this effect (Figure 3B).

Summary data from all animals revealed the same pattern of effects. Overall, M1 LFPs during TJM epochs (red) exhibited significantly more power in the 3–7 Hz range as compared to no TJM (blue) epochs (Figure 3C, left; *t*_(18)_ = 5.13, *p* < 0.05). The same pattern of activity existed for STN recording sites (Figure 3C, right; *t*_(18)_ = 4.55, *p* < 0.05). Further, M1 exhibited more power in the tremor band during TJM and no TJM epochs compared to STN (TJM: *t*_(18)_ = 3.47, *p* < 0.05; no TJM: *t*_(18)_ = 3.26, *p* < 0.05). Analysis of centroid frequency (Figure 3D) revealed no differences between bouts of TJMs and no TJMs within a given brain area (e.g., M1 TJM vs. M1 no TJM; M1: *t*_(18)_ = −0.79, *p* > 0.05; STN: *t*_(18)_ = −0.36, *p* > 0.05). Moreover, there were no significant differences in LFP centroid frequency between TJM and no TJM epochs across brain areas (e.g., M1 TJM vs. STN TJM; TJM: *t*_(18)_ = 0.51, *p* > 0.05; No TJM: *t*_(18)_ = 1.88, *p* > 0.05).

### Beta Band Power does not Increase during TJMs in M1 and STN

**Figure 4:**
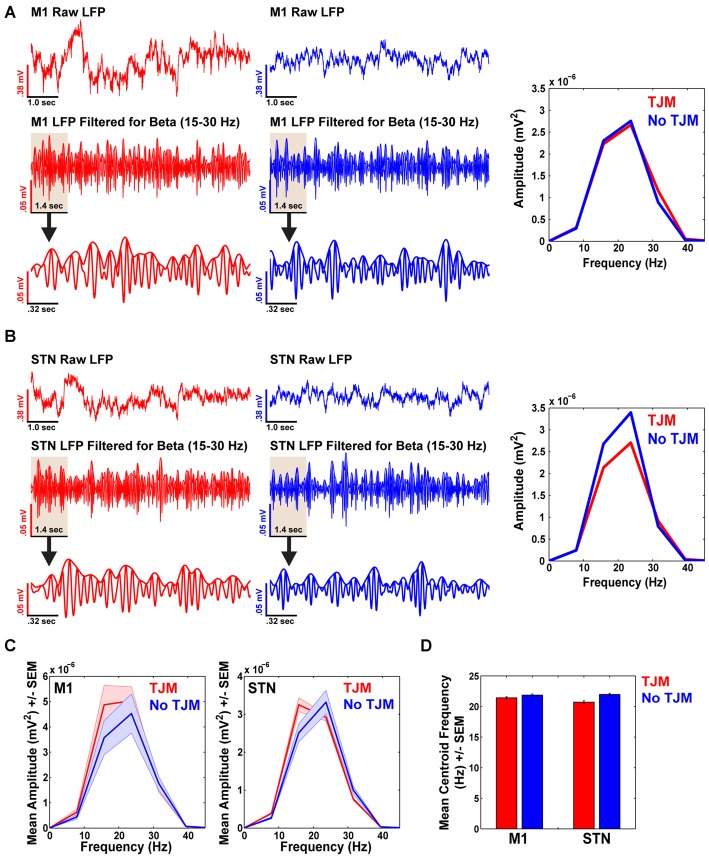
**Beta band power does not increase during TJMs in M1 and STN. (A)** Raw (top) and bandpass filtered (15–30 Hz; middle) M1 LFP traces during epochs of TJMs (red) and no TJMs (blue) for the same representative animal presented in Figures 2, 3. (**A**, bottom): The first 1.4 s of the middle panel of **(A)** to show instantaneous LFP fluctuations. (**A**, right): Power spectrum of the bandpass filtered LFP signal presented in the middle panel of **(A)** during the TJM and no TJM epoch. Beta frequency power does not increase during periods of TJMs. **(B)** Same as **(A)**, but for a simultaneously recorded STN site for the same representative animal presented in **(A). (C)** Summary data for beta band power across all animals for TJM and no TJM epochs for M1 and STN. As can be seen, beta band power is similar across behavioral states. **(D)** Summary data across all animals for beta band centroid frequency as a function of TJM and no TJM epochs for M1 and STN recording sites.

Simultaneously recorded M1 LFPs for the animal shown in Figures 2, 3, revealed similar LFP beta band power during epochs of TJMs (Figure 4A left, red) and epochs of quiescence (Figure 4A right, blue) for the raw (Figure 4A top) and bandpass filtered (15–30 Hz; Figure 4A middle/bottom) LFP signals. Importantly, the data shown here are for the same time points as presented in Figures 2, 3, but here data were filtered for beta (15–30 Hz) instead of tremor frequencies (3–7 Hz). A closer look at the signal revealed similar instantaneous fluctuations in the LFP during active (Figure 4A bottom, left) and quiet (Figure 4A bottom, right) bouts. The power spectrum of the raw LFP signal filtered for beta band activity during epochs of TJMs (red) and no TJMs (blue) revealed strong LFP beta band power, but no alterations in power across behavioral state (e.g., bouts of TJMs vs. no TJMs; Figure 4A, right). At a simultaneously recorded STN site, the same pattern of activity was present (Figure 4B). Overall, the bandpass filtered LFP signal (15–30 Hz) and subsequent power spectrum of that signal during epochs of TJMs and quiescence revealed no differences in beta band power (Figure 4B).

Summary data from all animals demonstrate the same trend as represented in Figures 4A,B. Overall, M1 LFPs during TJM epochs (red) and no TJM epochs (blue) exhibited similar levels of beta band power (Figure 4C; *t*_(18)_ = 1.48, *p* > 0.05). For STN, the same pattern of activity existed (Figure 4C, right; *t*_(18)_ = 0.65, *p* > 0.05). Further, M1 exhibited higher beta band power during epochs of TJMs and no TJMs as compared to STN (TJM: *t*_(18)_ = 2.76, *p* < 0.05; no TJM: *t*_(18)_ = 3.12, *p* < 0.05). Moreover, TJM epochs exhibited lower centroid beta band frequency compared to no TJM epochs for both M1 and STN (Figure 4D; M1: *t*_(18)_ = −2.84, *p* < 0.05; STN: *t*_(18)_ = −3.57, *p* < 0.05, respectively). Further, beta band frequency was higher in M1 during TJM epochs compared to STN (*t*_(18)_ = 3.63, *p* < 0.05). Alternatively, there was no difference in beta band frequency between M1 and STN during periods of quiescence (*t*_(18)_ = −0.47, *p* > 0.05).

## Discussion

Pilocarpine is a well known tremorogenic agent, and previous studies have shown that pilocarpine-induced TJMs are reduced by antiparkinsonian drugs (Salamone et al., 2005; Betz et al., 2009; Collins et al., 2010; Podurgiel et al., 2013a), conditional neural knockout of adenosine A_2A_ receptors (Salamone et al., 2013), and deep brain stimulation of the STN (Collins-Praino et al., 2013). Pilocarpine-induced TJMs are central in origin, and depend upon stimulation of M2 or M4 muscarinic receptors in the ventrolateral neostriatum of the rat, which is the homolog of the ventral putamen of primates (Mayorga et al., 1997, 1999; Salamone et al., 1998). Pilocarpine was employed in the present studies in order to induce a robust TJM response that would allow for the assessment of tremor-related EMG activity recorded simultaneously with cortical and subthalamic LFP activity. The temporalis muscle was used for the EMG measurements because of previous research indicating that activity of this jaw closing muscle is a critical marker of observable TJM activity (Cousins et al., 1998). As described above, the induction of TJMs by pilocarpine was associated with strong rhythmicity in the envelope of the temporalis EMG signal, with a peak frequency of approximately 4 Hz, along with robust second and third harmonics (Figure 2). This is consistent with EMG recordings from the forearms of PD patients during periods of tremor (Liu et al., 2002; Timmermann et al., 2003; Wang et al., 2005; Reck et al., 2009; Hirschmann et al., 2013).

These data are consistent with previous results from both clinical and preclinical studies, which indicate that cholinomimetic-induced TJMs share characteristics with human Parkinsonian tremor. Cholinomimetic drugs, including muscarinic agonists and anticholinesterases, can induce or exacerbate resting tremor in humans. Administration of the anticholinesterase physostigmine to PD patients was reported to exacerbate Parkinsonian symptoms, including tremor, and these motor deficits were attenuated by co-administration of centrally-acting muscarinic antagonists (Duvoisin, 1967). For many decades, muscarinic receptor antagonists have been used to treat idiopathic and drug-induced Parkinsonism (McEvoy, 1983). Anticholinesterases are prescribed to treat the cognitive deficits associated with Alzheimer’s Disease (for review, see Birks, 2006), and these drugs have been shown to induce or exacerbate Parkinsonian symptoms, including tremor, as side effects (Ott and Lannon, 1992; Keltner, 1994; Shea et al., 1998; Arai, 2000; Aarsland et al., 2003; Gurevich et al., 2006; Song et al., 2008). In rodent studies, muscarinic agonists and anticholinesterases induce TJMs, and co-administration of antiparkinsonian agents including DA agonists, L-DOPA, muscarinic antagonists, and adenosine A_2A_ antagonists have been shown to reduce cholinomimetic-induced TJMs (Mayorga et al., 1997; Salamone et al., 1998, 2013, 2015a,b; Simola et al., 2004, 2006; Miwa et al., 2009; Collins et al., 2010; Collins-Praino et al., 2011; Yasuda et al., 2015).

In the present studies, tremor-related EMG activity was accompanied by an increase in power at tremor frequency (3–7 Hz) in M1 and the STN (Figure 3). In PD patients, pathological oscillatory neuronal activity in the open and closed loop connections between the cortex, basal ganglia, and thalamus is thought to underlie tremorogenesis (Hutchison et al., 2004). The development of tremor has been associated with the emergence of oscillations in the tremor frequency range (3–7 Hz) as indicated by power spectra of STN LFPs, and coherence between STN LFPs and EMG activity at tremor frequency (Levy et al., 2000; Brown et al., 2001; Liu et al., 2002; Wang et al., 2005; Reck et al., 2009; Hirschmann et al., 2013; Oswal et al., 2013). In a recent study (Hirschmann et al., 2013), the emergence of tremor in PD patients was shown to be associated with an increase of cerebral synchronization at tremor frequency and the second harmonic in a network that includes both STN and M1. In African green monkeys treated with 1-methyl-4-phenyl-1,2,3,6-tetrahydropyridine (MPTP), the development of resting tremor was associated with the emergence of oscillations at tremor frequency in the STN (Bergman et al., 1994). Furthermore, previous studies have observed a strong coherence between EMG of forearm muscles and M1 activity at tremor frequency and its second harmonic (Timmermann et al., 2003).

In contrast to the observed association between TJM-related EMG recordings and LFP activity in the tremor frequency range (Figure 3), tremor activity was not specifically associated with increased synchrony in the beta frequency band in either M1 or STN (Figure 4). Oscillatory activity in the STN of PD patients has been well characterized, as researchers are able to record LFPs from patients undergoing implantation of electrodes for deep brain stimulation. In recent years, considerable focus has been on increased oscillatory activity in the beta band (~15–30 Hz), since there is evidence that increased beta power in the STN is associated with motor control, particularly akinesia-rigidity (Brown et al., 2001; Levy et al., 2002; Priori et al., 2004; Brown and Williams, 2005; Kühn et al., 2006). Nevertheless, resting tremor has not been shown to correlate with beta band activity in the STN of PD patients (Kühn et al., 2006). Thus, the results from our experiments in rats are line with the findings reported in the clinical and non-human primate studies, as we observed increased power in the tremor frequency range in M1 and STN during periods of tremor activity, but did not observe increased power in the beta frequency range during periods of TJMs.

The present results are consistent with previous studies demonstrating that the STN is a critical part of the basal ganglia circuitry that is involved in motor dysfunctions related to Parkinsonism. STN neurons were reported to show alterations in firing pattern and hyperactivity in Parkinsonian primates treated with MPTP (Miller and DeLong, 1987). In human PD patients, synchronized high-frequency (i.e., 15–30 Hz) oscillations have been recorded from the STN (Levy et al., 2000). STN high-frequency stimulation has been shown to improve akinesia, rigidity, postural and gait instabilities, and tremor in patients with PD (Limousin et al., 1995; Deuschl et al., 2006; Moro et al., 2010). Moreover, L-DOPA was reported to enhance the antiparkinsonian efficacy of STN stimulation (Bejjani et al., 2000). Lesions or inactivation of STN have been shown to reverse motor dysfunctions in rodent models (Centonze et al., 2005; Baunez and Gubellini, 2010). In addition, high frequency stimulation of STN has been reported to restore motor function in rodent models of Parkinsonism (Baunez, 2011; Brown et al., 2011), and to attenuate drug-induced TJMs in rats (Collins-Praino et al., 2013).

Network activation as measured by LFP activity in M1 and STN can be used as a tool to better understand transient dynamics across distributed neural networks. For example, abnormalities in long-range connectivity between brain areas have been postulated as important pathophysiological mechanisms underlying brain dysfunctions (Hutchison et al., 2004; Mallet et al., 2008). Detailed analysis of signals such as the LFP and EEG can reveal the engagement of distributed neural circuits in relation to tremorogenesis. However, it remains unclear how perturbed connectivity relates to motor symptoms such as tremorogenesis, and how it is manifested in the dynamic interactions of neuronal circuits. For that reason, it is important to study tremor-related oscillatory activity by using animal models that allow for the investigation of the specific circuits and neural mechanisms involved. Although oscillatory neural activity has been well documented in the clinical literature related to Parkinsonism, very little has been done to study these oscillatory alterations in rodent models. LFPs recorded from the frontal cortex and STN of rats with 6-OHDA lesions of midbrain dopaminergic neurons show increased power and coherence in the beta frequency band (Sharott et al., 2005; Mallet et al., 2008). However, it should be noted that these studies were using a model that involved neurotoxic depletion of DA, and were not specifically designed to focus on tremor-related activity. In contrast, our study employed a model of drug-induced Parkinsonian resting tremor; though the pilocarpine-treated rats appeared to have reduced locomotion after pilocarpine administration, they were not completely akinetic, and occasionally moved about the chamber during recording. Therefore, our study specifically evaluated the physiological correlates of tremor by using an agent that induces a robust tremorogenic (i.e., TJM) response.

In summary, pilocarpine-induced TJMs, which share several characteristics with Parkinsonian tremor (Salamone et al., 1998; Collins-Praino et al., 2011), are accompanied by rhythmic oscillatory neural activity in the tremor frequency range in both STN and M1. In view of the fact that the TJM model is frequently used to characterize novel drugs for their ability to suppress tremor (Collins et al., 2010, 2012; Santerre et al., 2012; Podurgiel et al., 2013a), it has been suggested that the combination of physiological and behavioral measures of tremor could be employed in preclinical studies focused on characterizing the neural circuitry that underlies tremorogenesis, and for the assessment of treatments that specifically target tremor (Salamone et al., 2015a).

## Author Contributions

JDS supervised the research project, and is the principle investigator. LLL and SJP contributed equally to this manuscript, and conducted the experiments and the analyses. AFH and ELE assisted with the performance of the experiments. JJC supervised the electrophysiological recordings. All authors contributed to writing the manuscript.

## Funding

This work was supported by a grant to JDS from the University of Connecticut Research Foundation, SURF grants to AFH and ELE, and a University of Connecticut Honors Life Sciences Thesis Award to ELE. SJP was supported by a grant from the Parkinson’s Disease Foundation.

## Conflict of Interest Statement

The authors declare that the research was conducted in the absence of any commercial or financial relationships that could be construed as a potential conflict of interest.

## Supplementary Material

FIGURE S1 **Verification of electrode placements across all animals. (A)** M1 electrode placement termination points (red dots) across five rats (rows). **(B)** Same as for **(A)**, but for STN electrodes. Each animal simultaneously contributed 3–4 M1 and STN placements (Rat one contributed three electrodes for each areal region, while all other animals contributed 4) for a total of 19 M1 electrodes and 19 STN electrodes across five animals (*n* = 38). M1 placements ranged from AP: +0.72 to +2.28 mm (relative to Bregma), while STN placements ranged from AP: −3.24 to +3.96 mm (relative to Bregma).

## References

[B1] Aarsland D. Hutchison M. Larsen J. P. (2003). Cognitive, psychiatric and motor response to galantamine in Parkinson’s disease with dementia. Int. J. Geriatr. Psychiatry 18, 937–941. 10.1002/gps.949 14533126

[B2] Arai M. (2000). Parkinsonism onset in a patient concurrently using tiapride and donepezil. Intern. Med. 39:863. 10.2169/internalmedicine.39.863 11030219

[B3] Baunez C. (2011). A few examples of the contribution of animal research in rodents for clinical application of deep brain stimulation. Prog. Brain Res. 194, 105–116. 10.1016/B978-0-444-53815-4.00013-3 21867798

[B4] Baunez C. Gubellini P. (2010). Effects of GPi and STN inactivation on physiological, motor, cognitive and motivational processes in animal models of Parkinson’s disease. Prog. Brain Res. 183, 235–258. 10.1016/s0079-6123(10)83012-2 20696323

[B104] Bejjani B. P. Gervais D. Arnulf I. Papadopoulos S. Demeret S. Bonnet A. M.. (2000). Axial parkinsonian symptoms can be improved: the role of levodopa and bilateral subthalamic stimulation. J. Neurol. Neurosurg. Psychiatry 68, 595–600. 10.1136/jnnp.68.5.595 10766889 PMC1736917

[B103] Bergman H. Wichmann T. Karmon B. DeLong M. R. (1994). The primate subthalamic nucleus. II. Neuronal activity in the MPTP model of parkinsonism. J. Neurophysiol. 72, 507–520. 7983515 10.1152/jn.1994.72.2.507

[B100] Betz A. J. Vontell R. Valenta J. Worden L. Sink K. S. Font L. (2009). Effects of adenosine A2A antagonist KW-6002 (istradefylline) on pimozide-induced oral tremor and striatal c-Fos expression: Comparisons with the muscarinic antagonist tropicamide. Neuroscience 163, 97–108. 10.1016/j.neuroscience.2009.05.040 19467297

[B5] Birks J. S. (2006). Cholinesterase inhibitors for Azheimer’s disease. Cochrane Database Syst. Rev. 1:CD005593. 10.1002/14651858.CD005593 16437532 PMC9006343

[B7] Brown P. (2003). Oscillatory nature of human basal ganglia activity: relationship to the pathophysiology of Parkinson’s disease. Mov. Disord. 18, 357–363. 10.1002/mds.10358 12671940

[B6] Brown A. R. Antle M. C. Hu B. Teskey G. C. (2011). High frequency stimulation of the subthalamic nucleus acutely rescues motor deficits and neocortical movement representations following 6-hydroxydopamine administration in rats. Exp. Neurol. 231, 82–90. 10.1016/j.expneurol.2011.05.017 21683073

[B8] Brown P. Oliviero A. Mazzone P. Insola A. Tonali P. Di Lazzaro V. (2001). Dopamine dependency of oscillations between subthalamic nucleus and pallidum in Parkinson’s disease. J. Neurosci. 21, 1033–1038. 11157088 10.1523/JNEUROSCI.21-03-01033.2001 PMC6762327

[B9] Brown P. Williams D. (2005). Basal ganglia local field potential activity: character and functional significance in the human. Clin. Neurophysiol. 116, 2510–2519. 10.1016/j.clinph.2005.05.009 16029963

[B10] Centonze D. Gubellini P. Rossi S. Picconi B. Pisani A. Bernardi G.. (2005). Subthalamic nucleus lesion reverses motor abnormalities and striatal glutamatergic overactivity in experimental parkinsonism. Neuroscience 133, 831–840. 10.1016/j.neuroscience.2005.03.006 15893432

[B11] Collins L. E. Galtieri D. J. Brennum L. T. Sager T. N. Hockemeyer J. Müller C. E.. (2010). Oral tremor induced by the muscarinic agonist pilocarpine is suppressed by the adenosine A2A antagonists MSX-3 and SCH58261, but not the adenosine A1 antagonist DPCPX: possible relevance for drug-induced parkinsonism. Pharmacol. Biochem. Behav. 94, 561–569. 10.1016/j.pbb.2009.11.011 19958787

[B12] Collins L. E. Sager T. N. Sams A. G. Pennarola A. Port R. G. Shahriari M.. (2012). The novel adenosine A2A antagonist Lu AA47070 reverses the motor and motivational effects produced by dopamine D2 receptor blockade. Pharmacol. Biochem. Behav. 100, 498–505. 10.1016/j.pbb.2011.10.015 22037410

[B13] Collins-Praino L. E. Paul N. E. Ledgard F. Podurgiel S. J. Kovner R. Baqi Y.. (2013). Deep brain stimulation of the subthalamic nucleus reverses oral tremor in pharmacological models of parkinsonism: interaction with the effects of adenosine A2A antagonism. Eur. J. Neurosci. 38, 2183–2191. 10.1111/ejn.12212 23600953

[B14] Collins-Praino L. E. Paul N. E. Rychalsky K. L. Hinman J. R. Chrobak J. J. Senatus P. B.. (2011). Pharmacological and physiological characterization of the tremulous jaw movement model of parkinsonian tremor: potential insights into the pathophysiology of tremor. Front. Syst. Neurosci. 5:49. 10.3389/fnsys.2011.00049 21772815 PMC3131529

[B15] Cousins M. S. Atherton A. Salamone J. D. (1998). Behavioral and electromyographic characterization of the local frequency of tacrine-induced tremulous jaw movements. Physiol. Behav. 64, 153–158. 10.1016/s0031-9384(98)00021-3 9662079

[B16] Cousins M. S. Carriero D. L. Salamone J. D. (1997). Tremulous jaw movements induced by the acetylcholinesterase inhibitor tacrine: effects of antiparkinsonian drugs. Eur. J. Pharmacol. 322, 137–145. 10.1016/s0014-2999(97)00008-3 9098680

[B17] Deuschl G. Schade-Brittinger C. Krack P. Volkmann J. Schäfer H. Bötzel K.. (2006). A randomized trial of deep-brain stimulation for Parkinson’s disease. N. Engl. J. Med. 355, 896–908. 10.1056/NEJMoa060281 16943402

[B18] Duvoisin R. C. (1967). Cholinergic-anticholinergic antagonism in parkinsonism. Arch. Neurol. 17, 124–136. 10.1001/archneur.1967.00470260014002 4382112

[B19] Gandía J. Morató X. Stagljar I. Fernández-Dueñas V. Ciruela F. (2015). Adenosine A2A receptor-mediated control of pilocarpine-induced tremulous jaw movements is Parkinson’s disease-associated GPR37 receptor-dependent. Behav. Brain Res. 288, 103–106. 10.1016/j.bbr.2015.04.001 25862943

[B20] George J. S. Strunk J. Mak-McCully R. Houser M. Poizner H. Aron A. R. (2013). Dopaminergic therapy in Parkinson’s disease decreases cortical beta band coherence in the resting state and increases cortical beta band power during executive control. Neuroimage Clin. 3, 261–270. 10.1016/j.nicl.2013.07.013 24273711 PMC3814961

[B21] Gurevich T. Y. Shabtai H. Korczyn A. D. Simon E. S. Giladi N. (2006). Effect of rivastigmine on tremor in patients with Parkinson’s disease and dementia. Mov. Disord. 21, 1663–1666. 10.1002/mds.20971 16941467

[B22] Hammond C. Bergman H. Brown P. (2007). Pathological synchronization in Parkinson’s disease:networks, models and treatments. Trends Neurosci. 30, 357–364. 10.1016/j.tins.2007.05.004 17532060

[B23] Herrera-Meza G. Manzo J. Hernández M. E. Miquel M. García L. I. (2014). Induction of mandibular tremor using electrolytic lesion of the ventrolateral striatum or using subchronic haloperidol therapy in male rats: an electromyographic comparison. Neurologia 29, 416–422. 10.1016/j.nrl.2013.10.003 24332783

[B24] Hirschmann J. Hartmann C. J. Butz M. Hoogenboom N. Ozkurt T. E. Elben S.. (2013). A direct relationship between oscillatory subthalamic nucleus-cortex coupling and rest tremor in Parkinson’s disease. Brain 136, 3659–3670. 10.1093/brain/awt271 24154618

[B25] Hornykiewicz O. (1973). Dopamine in the basal ganglia. Its role and therapeutic implications (including the clinical use of L-DOPA). Br. Med. Bull. 29, 172–178. 4356552 10.1093/oxfordjournals.bmb.a070990

[B102] Hutchison W. D. Dostrovsky J. O. Walters J. R. Courtemanche R. Boraud T. Goldberg J.. (2004). Neuronal oscillations in the basal ganglia and movement disorders: evidence from whole animal and human recordings. J. Neurosci. 24, 9240–9243. 10.1523/jneurosci.3366-04.2004 15496658 PMC6730107

[B27] Jicha G. Salamone J. D. (1991). Vacuous jaw movements and feeding deficits in rats with ventrolateral striatal dopamine depletions: possible model of parkinsonian symptoms. J. Neurosci. 11, 3822–3829. 1744692 10.1523/JNEUROSCI.11-12-03822.1991 PMC6575267

[B28] Keltner N. L. (1994). Tacrine: a pharmacological approach to Alzheimer’s disease. J. Psychosoc. Nurs. Ment. Health Serv. 32, 37–39. 10.1097/00002093-199424000-00005 8196019

[B29] Kühn A. A. Kupsch A. Schneider G. H. Brown P. (2006). Reduction in subthalamic 8–35 Hz oscillatory activity correlates with clinical improvement in Parkinson’s disease. Eur. J. Neurosci. 23, 1956–1960. 10.1111/j.1460-9568.2006.04717.x 16623853

[B30] Kühn A. A. Trottenberg T. Kivi A. Kupsch A. Schneider G. H. Brown P. (2005). The relationship between local field potential and neuronal discharge in the subthalamic nucleus of patients with Parkinson’s disease. Exp. Neurol. 194, 212–220. 10.1016/j.expneurol.2005.02.010 15899258

[B31] Levy R. Ashby P. Hutchison W. D. Lang A. E. Lozano A. M. Dostrovsky J. O. (2002). Dependence of subthalamic nucleus oscillations on movement and dopamine in Parkinson’s disease. Brain 125, 1196–1209. 10.1093/brain/awf128 12023310

[B32] Levy R. Hutchison W. D. Lozano A. M. Dostrovsky J. O. (2000). High-frequency synchronization of neuronal activity in the subthalamic nucleus of parkinsonian patients with limb tremor. J. Neurosci. 20, 7766–7775. 11027240 10.1523/JNEUROSCI.20-20-07766.2000 PMC6772896

[B33] Limousin P. Pollak P. Benazzouz A. Hoffmann D. Broussolle E. Perret J. E.. (1995). Bilateral subthalamic nucleus stimulation for severe Parkinson’s disease. Mov. Disord. 10, 672–674. 10.1002/mds.870100523 8552123

[B34] Liu X. Ford-Dunn H. L. Hayward G. N. Nandi D. Miall R. C. Aziz T. Z.. (2002). The oscillatory activity in the Parkinsonian subthalamic nucleus investigated using the macro-electrodes for deep brain stimulation. Clin. Neurophysiol. 113, 1667–1672. 10.1016/s1388-2457(02)00256-0 12417218

[B35] Mallet N. Pogosyan A. Sharott A. Csicsvari J. Bolam J. P. Brown P.. (2008). Disrupted dopamine transmission and the emergence of exaggerated beta oscillations in subthalamic nucleus and cerebral cortex. J. Neurosci. 28, 4795–4806. 10.1523/JNEUROSCI.0123-08.2008 18448656 PMC6670450

[B36] Marsden C. Duvoisin R. Jenner P. Parkes J. Pycock C. Tars D. (1975). Relationship between animal models and clinical parkinsonism. Adv. Neurol. 9, 165–175. 1146651

[B37] Mayorga A. J. Carriero D. L. Cousins M. S. Gianutsos G. Salamone J. D. (1997). Tremulous jaw movements produced by acute tacrine administration: possible relation to parkinsonian side effects. Pharmacol. Biochem. Behav. 56, 273–279. 10.1016/s0091-3057(96)00225-0 9050085

[B38] Mayorga A. J. Cousins M. S. Conlan A. Gianutsos G. Salamone J. D. (1999). Characterization of the muscarinic receptor subtype mediating pilocarpine-induced tremulous jaw movements in rats. Eur. J. Pharmacol. 364, 7–11. 10.1016/s0014-2999(98)00811-5 9920179

[B39] McEvoy J. P. (1983). The clinical use of anticholinergic drugs as treatments for extrapyramidal side effects of neuroleptic drugs. J. Clin. Psychpharmacol. 3, 288–302. 10.1097/00004714-198310000-00004 6138370

[B40] Miller W. C. DeLong M. R. (1987). “Altered tonic activity of neurons in the globus palidus and subthalamic nucleus in the primate MPTP model of parkinsonism,” in The Basal Ganglia II, eds Carpenter M. B. Jayaraman A. (New York, NY: Plenum Press), 415–427.

[B41] Miwa H. Kubo T. Suzuki A. Kondo T. (2009). Effects of zonisamide on c-Fos expression under conditions of tacrine-induced tremulous jaw movements in rats: a potential mechanism underlying its anti-parkinsonian tremor effect. Parkinsonism Relat. Disord. 15, 30–35. 10.1016/j.parkreldis.2008.02.008 18693129

[B42] Moro E. Lozano A. M. Pollak P. Agid Y. Rehncrona S. Volkmann J.. (2010). Long-term results of a multicenter study on subthalamic and pallidal stimulation in Parkinson’s disease. Mov. Disord. 25, 578–586. 10.1002/mds.22735 20213817

[B43] Oswal A. Brown P. Litvak V. (2013). Synchronized neural oscillations and the pathophysiology of Parkinson’s disease. Curr. Opin. Neurol. 26, 662–670. 10.1097/WCO.0000000000000034 24150222

[B44] Ott B. R. Lannon M. C. (1992). Exacerbation of Parkinsonism by tacrine. Clin. Neuropharmacol. 15, 322–325. 10.1097/00002826-199208000-00008 1516077

[B45] Paxinos G. Watson C. (2014). The Rat Brain in Stereotaxic Coordinates. San Deigo, CA: Academic Press.

[B46] Podurgiel S. J. Collins-Praino L. E. Yohn S. Randall P. A. Roach A. Lobianco C.. (2013a). Tremorolytic effects of safinamide in animal models of drug-induced parkinsonian tremor. Pharmacol. Biochem. Behav. 105, 105–111. 10.1016/j.pbb.2013.01.015 23360954

[B48] Podurgiel S. J. Nunes E. J. Yohn S. E. Barber J. Thompson A. Milligan M.. (2013b). The vesicular monoamine transporter (VMAT-2) inhibitor tetrabenazine induces tremulous jaw movements in rodents: implications for pharmacological models of parkinsonian tremor. Neuroscience 250, 507–519. 10.1016/j.neuroscience.2013.07.008 23867769

[B47] Podurgiel S. J. Milligan M. N. Yohn S. E. Purcell L. J. Contreras-Mora H. M. Correa M.. (2015). Administration exacerbates oral tremor and striatal dopamine depletion in a rodent pharmacological model of Parkinsonism. Neuropsychopharmacology 40, 2240–2247. 10.1038/npp.2015.69 25759301 PMC4613615

[B49] Priori A. Foffani G. Pesenti A. Tamma F. Bianchi A. M. Pellegrini M.. (2004). Rhythm-specific pharmacological modulation of subthalamic activity in Parkinson’s disease. Exp. Neurol. 189, 369–379. 10.1016/j.expneurol.2004.06.001 15380487

[B50] Reck C. Florin E. Wojtecki L. Krause H. Groiss S. Voges J.. (2009). Characterisation of tremor-associated local field potentials in the subthalamic nucleus in Parkinson’s disease. Eur. J. Neurosci. 29, 599–612. 10.1111/j.1460-9568.2008.06597.x 19187268

[B51] Salamone J. D. Betz A. J. Ishiwari K. Felsted J. Madson L. Mirante B.. (2008a). Tremorolytic effects of adenosine A2A antagonists: implications for parkinsonism. Front. Biosci. 13, 3594–3605. 10.2741/2952 18508458

[B54] Salamone J. D. Ishiwari K. Betz A. J. Farrar A. M. Mingote S. M. Font L.. (2008b). Dopamine/adenosine interactions related to locomotion and tremor in animal models: possible relevance to parkinsonism. Parkinsonism Relat. Disord. 14, S130–S134. 10.1016/j.parkreldis.2008.04.017 18585081 PMC2806674

[B52] Salamone J. D. Carlson B. B. Rios C. Lentini E. Correa M. Wisniecki A.. (2005). Dopamine agonists suppress cholinomimetic-induced tremulous jaw movements in an animal model of Parkinsonism: tremorolytic effects of pergolide, ropinirole and CY 208–243. Behav. Brain Res. 156, 173–179. 10.1016/j.bbr.2004.05.019 15582103

[B53] Salamone J. D. Collins-Praino L. E. Pardo M. Podurgiel S. J. Baqi Y. Müller C. E.. (2013). Conditional neural knockout of the adenosine A2A receptor and pharmacological A2A antagonism reduce pilocarpine-induced tremulous jaw movements: studies with a mouse model of parkinsonian tremor. Eur. Neuropsychopharmacol. 23, 972–977. 10.1016/j.euroneuro.2012.08.004 22947264

[B55] Salamone J. D. Mayorga A. J. Trevitt J. T. Cousins M. S. Conlan A. Nawab A. (1998). Tremulous jaw movements in rats: a model of parkinsonian tremor. Prog. Neurobiol. 56, 591–611. 10.1016/s0301-0082(98)00053-7 9871939

[B56] Salamone J. D. Podurgiel S. Collins-Praino L. E. Correa M. (2015a). “Physiological and behavioral assessment of tremor in rodents,” in Movement Disorders Genetics and Models, ed. LeDoux M. S. (Amsterdam: Elsevier), 631–640.

[B57] Salamone J. D. Podurgiel S. Long L. L. Nunes E. J. Correa M. (2015b). “Dopamine/adenosine interactions related to tremor in animal models of Parkinsonism,” in The Adenosinergic System: A Non-Dopaminergic Target in Parkinson’s Disease, eds Morelli M. Simola N. Wardas J. LeDoux M. S. (Heidelberg: Springer), 149–162.

[B58] Santerre J. L. Nunes E. J. Kovner R. Leser C. E. Randall P. A. Collins-Praino L. E.. (2012). The novel adenosine A2A antagonist prodrug MSX-4 is effective in animal models related to motivational and motor functions. Pharmacol. Biochem. Behav. 102, 477–487. 10.1016/j.pbb.2012.06.009 22705392

[B59] Sharott A. Magill P. J. Harnack D. Kupsch A. Meissner W. Brown P. (2005). Dopamine depletion increases the power and coherence of beta-oscillations in the cerebral cortex and subthalamic nucleus of the awake rat. Eur. J. Neurosci. 21, 1413–1422. 10.1111/j.1460-9568.2005.03973.x 15813951

[B60] Shea C. MacKnight C. Rockwood K. (1998). Donepezil for treatment of dementia with Lewy bodies: a case series of nine patients. Int. Psychogerniatr. 10, 229–238. 10.1017/s1041610298005341 9785144

[B61] Simola N. Fenu S. Baraldi P. G. Tabrizi M. A. Morelli M. (2004). Blockade of adenosine A2A receptors antagonizes parkinsonian tremor in the rat tacrine model by an action on specific striatal regions. Exp. Neurol. 189, 182–188. 10.1016/j.expneurol.2004.05.027 15296848

[B62] Simola N. Fenu S. Baraldi P. G. Tabrizi M. A. Morelli M. (2006). Dopamine and adenosine receptor interaction as basis for the treatment of Parkinson’s disease. J. Neurol. Sci. 248, 48–52. 10.1016/j.jns.2006.05.038 16780890

[B64] Song I. U. Kim J. S. Ryu S. B. Lee S. B. An J. Y. Lee K. S. (2008). Donepezil-induced jaw tremor. Parkinsonism Relat. Disord. 14, 584–585. 10.1016/j.parkreldis.2008.01.003 18321753

[B65] Timmermann L. Gross J. Dirks M. Volkmann J. Freund H. J. Schnitzler A. (2003). The cerebral oscillatory network of parkinsonian resting tremor. Brain 126, 199–212. 10.1093/brain/awg022 12477707

[B66] Wang S. Y. Aziz T. Z. Stein J. F. Liu X. (2005). Time-frequency analysis of transient neuromuscular events: dynamic changes in activity of the subthalamic nucleus and forearm muscles related to the intermittent resting tremor. J. Neurosci. Methods 145, 151–158. 10.1016/j.jneumeth.2004.12.009 15922033

[B67] Welch P. D. (1967). The use of fast fourier transform for the estimation of power spectra: a method based on time averaging over short, modified periodograms. IEEE Trans. Audio Electroacoust. 15, 70–73. 10.1109/tau.1967.1161901

[B68] Yasuda K. Abe H. Koganemaru G. Ikeda T. Arimori K. Ishida Y. (2015). Pramipexole reduces parkinsonian tremor induced by pilocarpine infusion in the rat striatum. Pharmacol. Biochem. Behav. 131, 1–5. 10.1016/j.pbb.2015.01.012 25622778

