# Supplementary figures and images for: Subthalamic and Cortical Local Field Potentials Associated with Pilocarpine-Induced Oral Tremor in the Rat
Source: Front Behav Neurosci. 2016 Jun 17;10:123. doi: 10.3389/fnbeh.2016.00123 (PMC4911403; doi:10.3389/fnbeh.2016.00123)

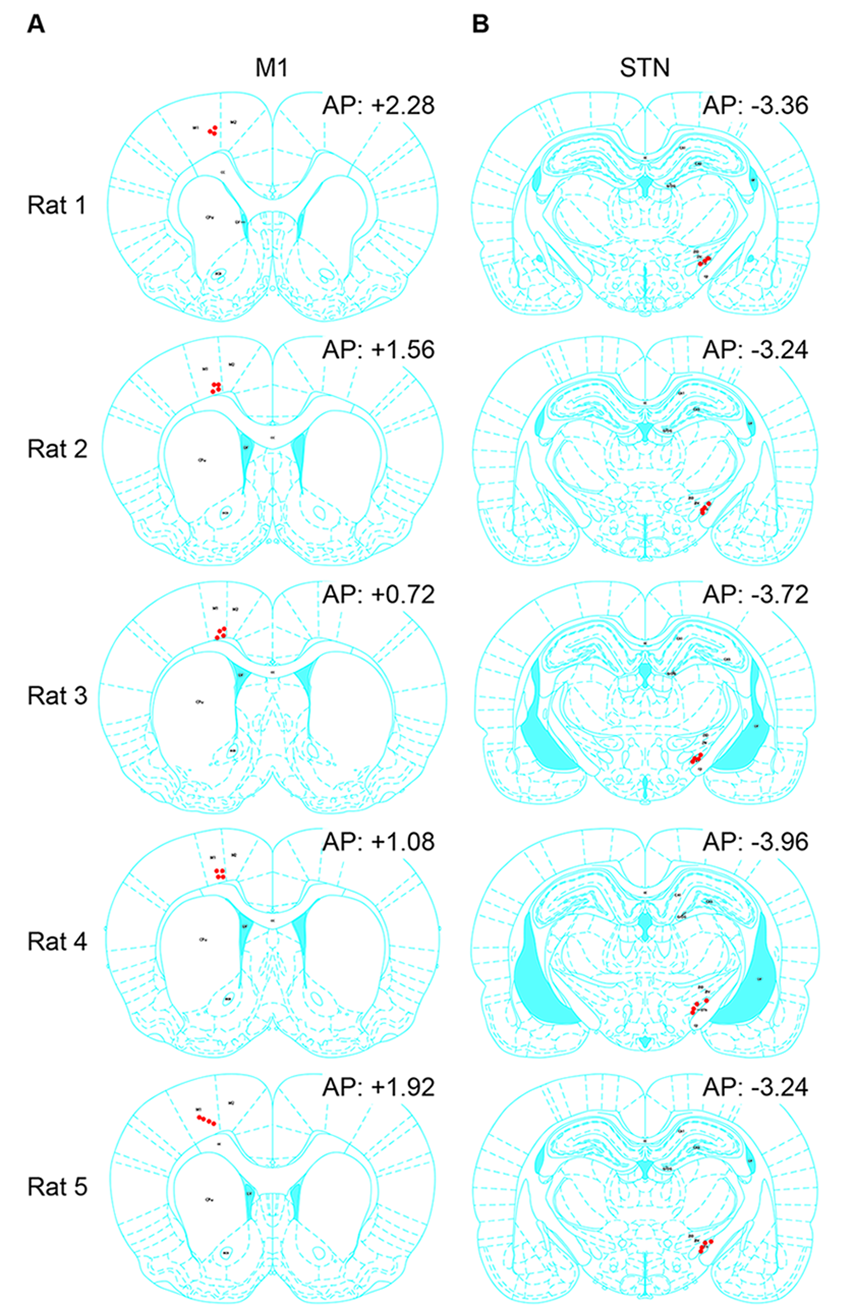

Supplement: FIGURE S1 — Verification of electrode placements across all animals. (A) M1 electrode placement termination points (red dots) across five rats (rows). (B) Same as for (A), but for STN electrodes. Each animal simultaneously contributed 3–4 M1 and STN placements (Rat one contributed three electrodes for each areal region, while all other animals contributed 4) for a total of 19 M1 electrodes and 19 STN electrodes across five animals (n = 38). M1 placements ranged from AP: +0.72 to +2.28 mm (relative to Bregma), while STN placements ranged from AP: −3.24 to +3.96 mm (relative to Bregma). [file Image_1.tif]
